# Injection Molding of Polymers and Polymer Composites

**DOI:** 10.3390/polym16131796

**Published:** 2024-06-25

**Authors:** Shengtai Zhou, Andrew N. Hrymak

**Affiliations:** 1 The State Key Laboratory of Polymer Materials Engineering, Polymer Research Institute of Sichuan University, Chengdu 610065, China; szhou@scu.edu.cn; 2 Department of Chemical and Biochemical Engineering, The University of Western Ontario, London, ON N6A 5B9, Canada

Injection molding technology has been widely adopted to fabricate multifunctional polymeric components or structural parts for applications in fields such as automotives, electronics, packaging, aerospace, and many others. It is also widely accepted that the properties of injection moldings are greatly affected by the development of crystalline structure, the distribution and orientation of functional fillers which are incurred by the prevailing shearing and extensional flow fields as well as the cooling effect during injection molding. In addition, the properties of polymeric parts are determined by the types of fillers and host matrices, part geometry, and processing parameters. Therefore, elucidating the relationship of processing–structure–properties in injection molding is particularly important for both the academic and industrial spheres.

The Special Issue, “Injection Molding of Polymers and Polymer Composites”, serves as a suitable platform for the state-of-the-art research progress in injection molding. This Special Issue collates 10 research articles, with contributions from Germany (1), China (4), the United States of America (1), Japan (2), Vietnam (1) and the Czech Republic (1), which covered a very broad range of topics relating to injection molding technology. The contributions to this Special Issue are listed below:

Contribution 1: Zhang, J.; Zhang, Y.; Li, Y.; Luo, M.; Zhang, J. Influence of Strong Shear Field on Structure and Performance of HDPE/PA6 In Situ Microfibril Composites. Polymers 2024, 16, 1032. https://doi.org/10.3390/polym16081032

Contribution 2: Bednarik, M.; Pata, V.; Ovsik, M.; Mizera, A.; Husar, J.; Manas, M.; Hanzlik, J.; Karhankova, M. The Modification of Useful Injection-Molded Parts’ Properties Induced Using High-Energy Radiation. Polymers 2024, 16, 450. https://doi.org/10.3390/polym16040450

Contribution 3: Minh, P.S.; Nguyen, V.-T.; Uyen, T.M.T.; Huy, V.Q.; Le Dang, H.N.; Nguyen, V.T.T. Enhancing Amplification in Compliant Mechanisms: Optimization of Plastic Types and Injection Conditions. Polymers 2024, 16, 394. https://doi.org/10.3390/polym16030394

Contribution 4: Tian, J.; Wang, C.; Wang, K.; Xue, R.; Liu, X.; Yang, Q. Flexible Polyolefin Elastomer/Paraffin Wax/Alumina/Graphene Nanoplatelets Phase Change Materials with Enhanced Thermal Conductivity and Mechanical Performance for Solar Conversion and Thermal Energy Storage Applications. Polymers 2024, 16, 362. https://doi.org/10.3390/polym16030362

Contribution 5: Jiang, Q.; Takayama, T.; Nishioka, A. Impact Energy Dissipation and Quantitative Models of Injection Molded Short Fiber-Reinforced Thermoplastics. Polymers 2023, 15, 4297. https://doi.org/10.3390/polym15214297

Contribution 6: Takayama, T.; Shibazaki, R. Mechanical Anisotropy of Injection-Molded PP/PS Polymer Blends and Correlation with Morphology. Polymers 2023, 15, 4167. https://doi.org/10.3390/polym15204167

Contribution 7: Myers, M.; Mulyana, R.; Castro, J.M.; Hoffman, B. Experimental Development of an Injection Molding Process Window. Polymers 2023, 15, 3207. https://doi.org/10.3390/polym15153207

Contribution 8: Yao, H.; Xue, R.; Wang, C.; Chen, C.; Xie, X.; Zhang, P.; Zhao, Z.; Li, Y. High-Temperature Response Polylactic Acid Composites by Tuning Double-Percolated Structures. Polymers 2023, 15, 138. https://doi.org/10.3390/polym15010138

Contribution 9: Zhou, C.; Bai, Y.; Zou, H.; Zhou, S. Improving Thermal Conductivity of Injection Molded Polycarbonate/Boron Nitride Composites by Incorporating Spherical Alumina Particles: The Influence of Alumina Particle Size. Polymers 2022, 14, 3477. https://doi.org/10.3390/polym14173477

Contribution 10: Maertens, R.; Liebig, W.V.; Weidenmann, K.A.; Elsner, P. Development of an Injection Molding Process for Long Glass Fiber-Reinforced Phenolic Resins. Polymers 2022, 14, 2890. https://doi.org/10.3390/polym14142890

The following provides an overview of the articles published in this Special Issue:

Zhang et al. (contribution 1) adopted a multi-flow vibration injection molding (MFVIM) technology to improve the mechanical properties of high-density polyethylene (HDPE) by tuning the crystalline structure of HDPE and creating polyamide 6 (PA) microfibers in situ. An HDPE/PA6 blend with a component mass ratio of 90:10 was used as the model system, and it was subjected to conventional injection molding (CIM, without vibration) and MFVIM, respectively. The tensile strength and tensile modulus of HDPE/PA6, which was prepared by implementing six vibration times during the packing stage, were 66.5 and 981.4 MPa, figures which are 91% and 32% higher than those for CIM pure HDPE, which were 83% and 27% higher than their CIM counterparts, respectively. The in situ formation of PA6 microfibers, as well as the numerous shish-kebab and hybrid shish-kebab structures of HDPE induced by the multiple shear zones generated by vibration, were considered as contributing factors.

Bednarik et al. (contribution 2) adopted a high energy radiation (β radiation) technique to modify the properties of injection-molded HDPE (representative commodity thermoplastic) and glass fiber (GF)-reinforced PA66 composites (GF content: 30 wt%, representative technical plastic). Their results show that the free surface energy of samples was altered, which was likely caused by oxidation, and this greatly affected the adhesive properties of the tested materials. In addition, the tensile and bending strength of both samples were enhanced after radiation treatment, which was attributed to the radiation induced cross-linking process. In the case of HDPE, an optimal dose was reported in a range from 145 to 150 kGry by taking both the surface and mechanical properties into account, and the optimal dose for PA66 was 128~135 kGry. They proposed that a well-chosen radiation dose leads to the improvement of both the mechanical and surface properties of injection-molded products that can broaden their practical applications.

Nguyen et al. (contribution 3) investigated the effect of process parameters such as filling time, filling pressure, filling speed, packing time, packing pressure, cooling time and melt temperature on the amplification ratio of the compliant mechanism injection molded flexure hinges made from ABS, PP, and HDPE. Their results demonstrated a linear relationship between the input and output data of ABS, PP, and HDPE flexure hinges at different process parameters. The packing pressure had the greatest impact on the amplification ratio of the ABS flexure hinge, filling time had the highest effect with a PP flexural hinge, and packing time had the greatest effect with HDPE flexural hinges. This work provides some insights to broaden the application of plastic flexure hinges by optimizing plastic types and injection-molding process parameters.

Yang et al. (contribution 4) prepared electrically insulative and thermally conductive polyolefin elastomer/paraffin wax (POE/PW) phase-change materials (PCMs) with spherical alumina (Al_2_O_3_) particles and graphene nanoplatelets (GNPs), using injection molding technique. The hybrid addition of Al_2_O_3_ and GNPs was found to be helpful for establishing three-dimensional thermal conductive pathways, thus improving thermal conductivity. The in-plane thermal conductivity of the POE/PW/GNPs 5 wt%/Al_2_O_3_ 40 wt% composite reached as high as 1.82 W/mK, which is approximately 269.5% higher than that of unfilled POE/PW. In addition, the POE/PW/GNPs 5 wt%/Al_2_O_3_ 40 wt% composite demonstrated outstanding electrical insulation, mechanical performance, and efficient solar energy conversion. This study showcased developing flexible PCMs for solar conversion and thermal storage applications.

Takayama et al. (contribution 5) proposed a mechanical model to explain the notched impact strength of injection-molded short glass-fiber-reinforced thermoplastics (SGFRTP). The model showed a good agreement (R^2^ > 0.95) with the experimental values obtained from GF-reinforced polypropylene (PP) and polystyrene (PS) composites. The authors suggested that the model could be applied to different fiber orientation angles and a range of fiber lengths in the molded products, provided that the fiber length was sufficiently shorter than the critical fiber length. They also stated that the universality of the proposed model needed to be verified if the fibers have weaker interfacial strength or less susceptibility to fracture during injection molding.

Takayama and Shibazaki (contribution 6) adopted a short-beam shear testing method to evaluate mechanical anisotropy as the stress concentration factor. The correlation between the evaluation results and the phase structure of PP/PS blends was clarified. They found that the yield condition under uniaxial tensile testing was interface debonding for the continuous-phase PP with a sea–island structure; the phase structure was dispersed and elongated in the flow direction for continuous-phase PS. Unlike continuous-phase PP, the structure of continuous-phase PS was greatly altered with the addition of styrene–ethylene–butadiene–styrene (SEBS). The yielding condition under uniaxial tensile loading was shear yielding. The development of the phase structure was affected by the types of PP, and the addition of SEBS to PS/H-PP (i.e., homo-type PP) resulted in a phase morphology with a cylindrical dispersed phase with relatively small diameter, and a dispersed phase arranged in a network for PS/B-PP (i.e., block-type PP). Moreover, the mechanical anisotropy of PP/PS blends was correlated with the aspect ratio of the dispersed phase. The higher the aspect ratio of the dispersed phase, the greater mechanical anisotropy for corresponding blends.

Castro et al. (contribution 7) studied the relationship between some key machine settings, which were classified as primary control variables (mold temperature, melt temperature, packing pressure), secondary control variables (injection screw speed, packing/cooling time), and tertiary control variables (shot size, clamping force) with the successful operation of injection molding. Their study provided a more standardized and thorough procedure for experimentally developing injection molding process windows to obtain injection-molded parts with acceptable appearance and part qualities. Additionally, it was proposed that testing the mechanical properties is a need for determining process windows for semi-crystalline polymers.

Zhao et al. (contribution 8) prepared electrically conductive polymers (CPCs) with a double-percolation structure consisting of poly(lactide acid) (PLA), poly(butylene adipate terephthalate) (PBAT), and GNPs. The results show that adding 5 wt% PBAT resulted in an electrical conductivity, which is about two orders of magnitude higher than the PLA/GNP 3.5 wt% counterpart. PBAT significantly reduced the action time from 14.15 to 2.19 min during temperature-response measurements. Moreover, PLA/PBAT/GNP samples showed more sensibility and stability during the cyclic temperature-response tests, which demonstrate potential applications for fabricating temperature-sensing devices.

Zhou et al. (contribution 9) systematically studied the effect of Al_2_O_3_ particle size and filling content on the properties of polycarbonate (PC)/boron nitride (BN) composites. They reported that both the in-plane (i.e., parallel to the flow direction) and through-plane (i.e., perpendicular to flow direction) thermal conductivities of injection-molded PC/BN/Al_2_O_3_ composites were significantly enhanced with the addition of Al_2_O_3_ particles. In addition, thermal conductivity was greatly improved with increasing Al_2_O_3_ concentration and particle size. The change in the orientation state of BN platelets that was induced by the added Al_2_O_3_ was crucial to improving through-plane thermal conductivity. Additionally, PC/BN/Al_2_O_3_ composites exhibit exceptional electrical insulation and reasonable mechanical properties that may provide potential applications in industrial sectors.

Maertens et al. (contribution 10) developed a long fiber direct thermoset injection molding process to prepare GF-reinforced phenolic resin composites with a higher proportion of long fibers, aiming to improve its mechanical properties. A novel screw-mixing element was adopted with considerations of balancing the desired mixing action, an undesired preliminary curing of phenolic resin, and a reduction in fiber length. The results show that a weighted average fiber length of 571 μm (initial fiber length: 5000 μm) was achieved in molded parts, which is twice that observed for a short fiber-reinforced phenolic resin under comparable processing conditions. In addition, a homogeneous distribution of fibers was found to outweigh the disadvantages of reduced fiber length because samples prepared with the highest mixing energy input had a tensile strength of 57 MPa, and the samples prepared with the lowest mixing energy input was only 21 MPa.

Finally, we would like to thank all researchers and anonymous reviewers who contributed to the production of this Special Issue. In addition, we express our sincere gratitude to the editorial team, especially Ms. Shelly Gu who contributed to the development and subsequent launch of this Special Issue.

## Data Availability

Data sharing is not applicable to this article.

